# Matrix metalloproteinases in coronary artery disease and myocardial infarction

**DOI:** 10.1007/s00395-023-00987-2

**Published:** 2023-05-09

**Authors:** Hanna Bräuninger, Saskia Krüger, Lucas Bacmeister, Alexander Nyström, Kilian Eyerich, Dirk Westermann, Diana Lindner

**Affiliations:** 1 Department of Cardiology and Angiology, University Heart Center Freiburg—Bad Krozingen, Medical Center—University of Freiburg, Faculty of Medicine, University of Freiburg, Hugstetter Str. 55, 79106 Freiburg, Germany; 2 German Centre for Cardiovascular Research (DZHK), Partner Side Hamburg/Kiel/Lübeck, Hamburg, Germany; 3 Clinic for Cardiology, University Heart and Vascular Centre Hamburg, Hamburg, Germany; 4 Department of Dermatology, Medical Center–University of Freiburg, Faculty of Medicine, University of Freiburg, Freiburg, Germany

**Keywords:** Matrix metalloproteinases, Myocardial infarction, Atherosclerosis, Single nucleotide polymorphisms, Single-cell sequencing data, Cardiac remodelling

## Abstract

Cardiovascular diseases (CVDs) remain the leading cause of death worldwide. Most cardiovascular deaths are caused by ischaemic heart diseases such as myocardial infarction (MI). Hereby atherosclerosis in the coronary arteries often precedes disease manifestation. Since tissue remodelling plays an important role in the development and progression of atherosclerosis as well as in outcome after MI, regulation of matrix metalloproteinases (MMPs) as the major ECM-degrading enzymes with diverse other functions is crucial. Here, we provide an overview of the expression profiles of MMPs in coronary artery and left ventricular tissue using publicly available data from whole tissue to single-cell resolution. To approach an association between MMP expression and the development and outcome of CVDs, we further review studies investigating polymorphisms in MMP genes since polymorphisms are known to have an impact on gene expression. This review therefore aims to shed light on the role of MMPs in atherosclerosis and MI by summarizing current knowledge from publically available datasets, human studies, and analyses of polymorphisms up to preclinical and clinical trials of pharmacological MMP inhibition.

## Introduction

Approximately 17.8 million deaths worldwide were attributable to cardiovascular diseases (CVDs) in 2017, making them the leading cause of death [36]. Thereby, most of these CVD-related deaths are caused by ischemic heart diseases including myocardial infarction (MI) [137]. This interruption of oxygen and nutrient supply to the heart is typically preceded by atherosclerosis in the coronary arteries [120]. Here, the rupture of unstable and vulnerable plaques can lead to coronary thrombus, causing type I MI due to coronary embolism [41, 44, 120]. The development and stability of atherosclerotic plaques is highly dependent on the abundance, composition, organization and integrity of their extracellular matrix (ECM), which includes proteins such as elastin and collagen fibrils. Destruction of these proteins promotes atherogenesis or destabilizes the fibrous cap of atherosclerotic plaques [96, 138]. ECM remodelling also plays a critical role in the outcome of MI: the cardiomyocyte death after MI is followed by reparative fibrosis replacing damaged tissue [31, 32]. Since adult cardiomyocytes are thought to be unable to proliferate to regenerate damaged myocardium [144], reparative fibrosis is essential to maintain cardiac integrity and to prevent left ventricular (LV) wall dilation. Yet, an exaggerated fibrotic response is at the same time detrimental by stiffening the cardiac tissue and needs to be mitigated [116]. Regarding the remodelling processes in atherosclerosis and MI, matrix metalloproteinases (MMPs), as a major class of enzymes with structural matrix protein-cleaving capabilities, have been in focus of cardiovascular research. The versatile family of MMPs consists of more than 20 members [54], which are involved in numerous biological processes including angiogenesis, embryonic development, tissue remodelling and growth, cell proliferation, migration, differentiation, and regulating immune responses [18]. Some of these actions are direct or associated consequences of MMP-mediated proteolysis of ECM proteins.

This review aims to provide an overview of the biological role of MMPs in the development and progression of atherosclerosis and MI deduced from publically available data. Thus, we provide an overview including expression data from human studies showing the diverse role of different MMPs in those remodelling processes. Moreover, we discuss the biological role of genetic polymorphisms in MMP genes in the context of atherosclerosis, MI development and post-MI remodelling.

## Structure, activation and function of MMPs

**Fig. 1:**
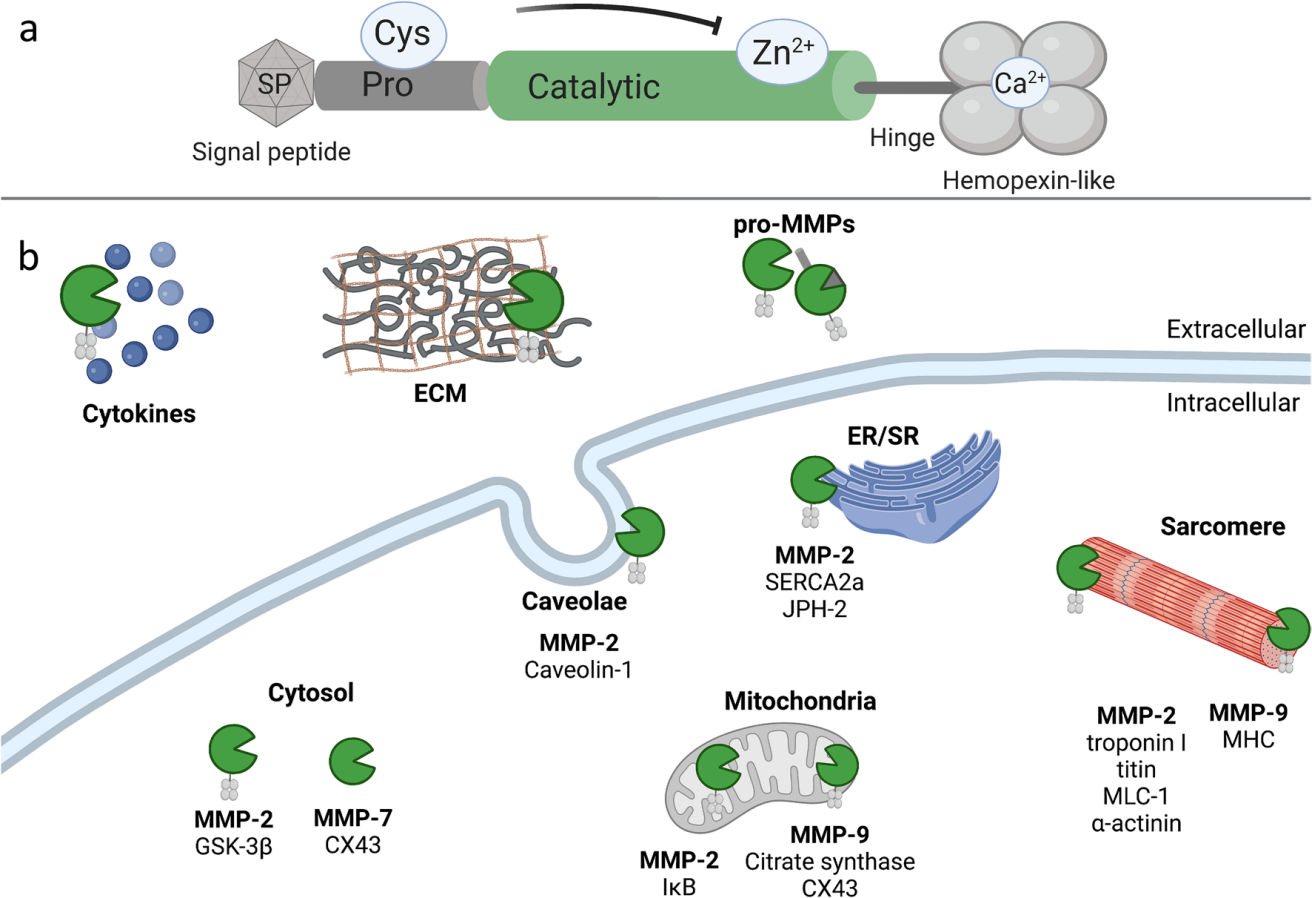
Structure of MMPs and summary of selected intra- and extracellular substrates in the cardiac context. (**a**) Most MMPs share a common structure consisting of a signal peptide (SP), a pro-peptide (Pro) with a conserved cysteine-residue (Cys), a catalytic domain binding a zinc (Zn^2+^) ion, a hinge region, and a hemopexin-like domain complexed with a calcium (Ca^2+^) ion. (**b**) In the past, only proteins of the extracellular matrix (ECM) were considered to be substrates of MMPs. Today it is known that MMPs have many extra- and intracellular substrates. Various MMPs extracellularly target cytokines and pro-MMPs in addition to ECM proteins. Intracellular target structures for MMP-2, -7, and -9 were selected and are depicted here. *CX43* connexin 43, *ER* endoplasmic reticulum, *GSK-3β* glycogen synthase kinase-3 beta, *IκB* inhibitor of kappa B-alpha, *JPH-2* junctophilin-2, *MLC-1* myosin light-chain I, *MHC* myosin heavy chain, *SERCA2a* Sarcoplasmic/endoplasmic reticulum calcium ATPase 2a, *SR* sarcoplasmic reticulum

In general, all MMPs share a common structure, which is listed below from N- to C-terminus. As depicted in Fig. 1a, they usually consist of a signal peptide, a pro-peptide, a catalytic domain, a hinge region, and a hemopexin-like domain. The signal peptide that targets the protein for secretion is usually 16–30 amino acids long and is cleaved off during translation. In general, MMPs are synthesized as zymogens and therefore they contain an 80 amino acids long N-terminal pro-domain. A cysteine residue within the pro-domain interacts with the central zinc (Zn^2+^) ion, which is attached to the catalytic domain, thus serving as an intra-molecular inhibitor [18]. The Zn^2+^-ion is attached to three conserved histidine residues within the zinc-binding motif of the 170 amino acids long catalytic domain [123]. The catalytic domain is linked by a hinge region of variable length to the hemopexin-like domain. This domain forms a four-blade-propeller structure, is slightly modified in some MMPs, and contributes to substrate specificity [18, 28, 115]. While MMP-7 and MMP-26 lack the hemopexin-like domain, membrane-bound MMPs (MT-MMPs) contain either an additional transmembrane type I domain or a glycosylphosphatidylinositol (GPI) anchor at the C-terminus [90].

Canonical, extracellular functions of MMPs are the degradation of ECM proteins, activation of cytokines and other pro-MMPs [18, 134]. In this case, the signal peptide targets the protein for secretion and the pro-domain is cleaved by proteolysis, activating the MMP [90]. Thus, MMPs are activated by the resolution of the cysteine-Zn^2+^ bond between the Pro- and catalytic domain. Nowadays, it is known that many MMPs are active not only in the extracellular space but also intracellularly, as previously comprehensively reviewed [6, 56]. Some MMPs remain inside the cell because of variations within their signal peptide sequence or re-enter the cell after secretion—in case of MMP-2 it was shown that almost half of the protein remains cytosolic [3, 50, 56]. For MMP-2, it is even known that there are two truncated splice forms lacking the signal peptide and are therefore not secreted [3, 77]. Several mechanisms for intracellular activation of MMPs have been identified. Among these, a major mechanism is activation by oxidative and nitrosative stress. However, modulation of MMP activity by phosphorylation and intracellular proteolytic activation has also been described [6, 50].

In the cardiac context, mainly intracellular functions of MMP-2 have been studied and reviewed so far [21, 50], but intracellular substrates of MMP-7 and MMP-9 have also been discovered in cardiomyocytes. Well-studied targets of MMP-2 are sarcomeric structures (troponin I, titin, myosin light-chain I (MLC-1)) and the cytoskeleton of cardiomyocytes (α-actinin) as depicted in Fig. 1 [2, 17, 109, 114, 122, 131]. These are degraded by MMP-2 under oxidative stress, particularly under ischemic conditions, which can lead to contractile dysfunction in the heart [2, 109, 114, 122, 131]. Furthermore, MMP-9 is thought to be involved in the degradation of sarcomere structures, in particular in myosin heavy chain [107]. In the endoplasmic reticulum (ER) of cardiomyocytes, MMP-2 contributes to the degradation of sarcoplasmic/ER calcium ATPase 2a and junctophilin-2 under ischemic conditions [16, 104]. Both MMP-2 and MMP-9 have been shown to target mitochondria-associated proteins. In cardiomyocytes, MMP-2 degrades the inhibitor of kappa B-alpha and MMP-9 targets mitochondrial citrate synthase and connexin 43 [19, 20, 76, 121]. MMP-7 has also been proven to degrade connexin 43 in MI, but in the cytosol rather than in mitochondria [72]. In the cytosol, also MMP-2 targets glycogen synthase kinase (GSK)-3β under oxidative stress, thereby increasing its kinase activity [61].

## Expression of MMPs in human tissues

**Fig. 2:**
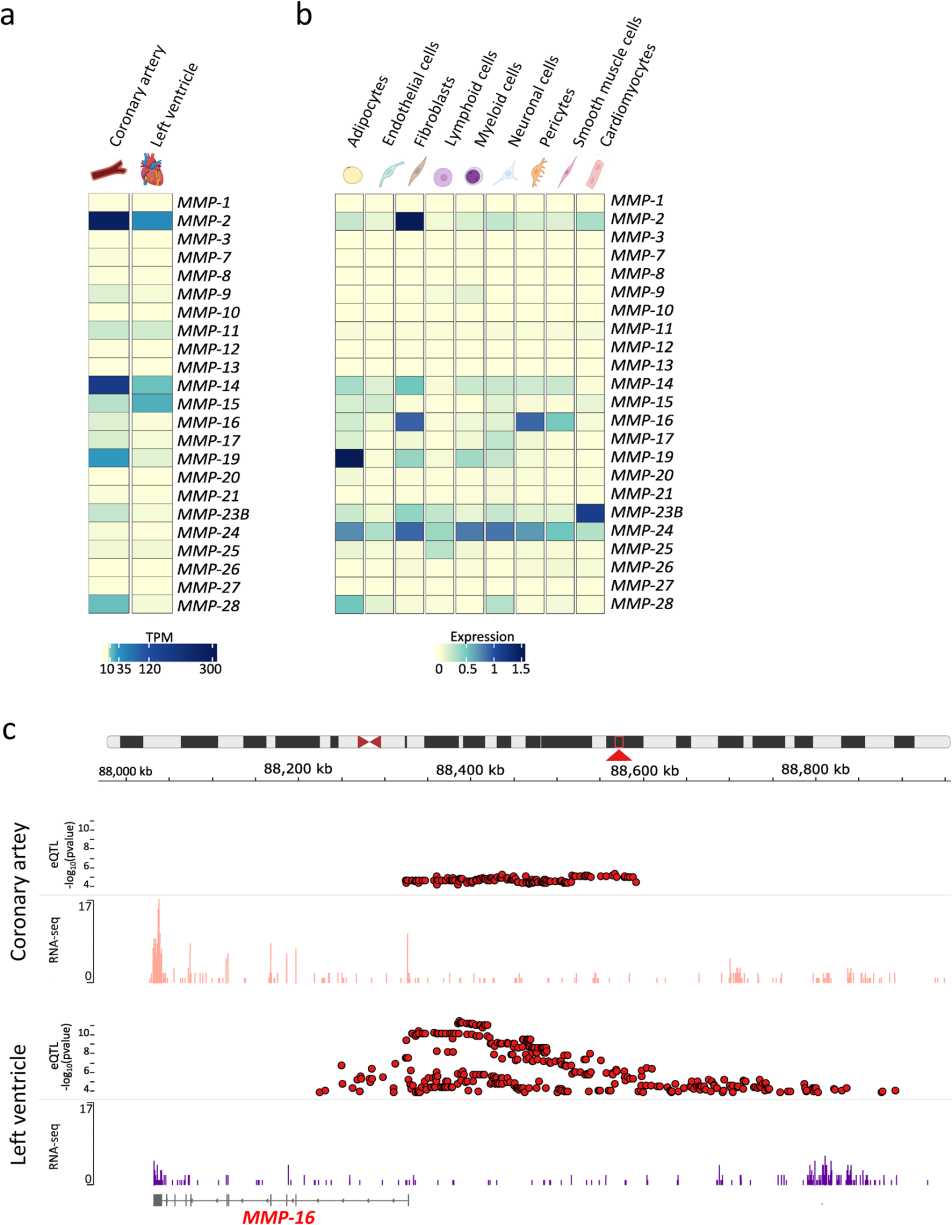
Gene expression of *MMPs* in human tissue. (**a**) Tissue-specific gene expression data as transcripts per million (TPM) from the GTEx project are plotted as heat map [39]. The expression of all human *MMPs* is compared between coronary artery and left ventricle. Since presumably non-diseased tissue-sites were sampled, *MMP* expression is relatively low. (**b**) Gene expression of all *MMPs* of different left-ventricular cell types from the single-cell sequencing dataset HeartCellAtlas is plotted as heat map [74]. (**c**) Since *MMP-16* is the only MMP in the GTEx database that has eQTLS in both, LV and coronary artery tissue, this example was selected to show how to obtain and depict information from the GTEx database. Using GTEx data, expression quantitative trait loci (eQTLs) and RNA-seq data are plotted using the integrative genomics viewer (IGV) for the *MMP-16* gene coded at chromosome 8 (red arrow). eQTLs, (red dots) are plotted for coronary artery (upper panel) and left ventricular tissue (lower panel) with the *p*-value (− log_10_) of eQTLs plotted on the *Y*-axis. Additionally, RNA-sequencing coverage from an individual sample is plotted for coronary artery (upper panel, light red) and left ventricular tissue (lower panel, purple) as histogram. The collapsed gene model of *MMP-16* is depicted below the RNA-sequencing coverage showing exon and intron structure of the gene. Data source: GTEx Analysis Release V8 [39] (dbGaP Accession phs000424.v8.p2) on 19.04.2021 created with GTEx IGV Browser [103, 119]

The human genome possesses 24 *MMP* genes, of which two genes encode an identical MMP-23 protein leading to 23 different MMPs. The Genotype-Tissue Expression (GTEx) project provides publically available, tissue-specific gene expression data from 54 tissue sites across nearly 1000 individuals [39]. For LV tissue samples, 432 donors were available, whereas for coronary artery tissue only 240 donors were included. The age range of the donors was 20–70 years. Unfortunately, GTEx does not report the cause of death for each donor individually. However, the majority of donors died of non-cardiac diseases. In Fig. 2a, the gene expression of all MMPs is plotted for LV and coronary tissue. Here, *MMP-2*, *-14* and *-19* are the highest expressed MMPs in coronary arteries and *MMP-2*, *-14* and *-15* in LV tissue. However, most MMPs are expressed at a low level in non-diseased tissue. Moreover, the recently published single-cell sequencing dataset HeartCellAtlas provides information about *MMP* expression in healthy human cardiac tissue on single-cell level [74]. Here, heart tissue from 14 adult donors was processed. In Fig. 2b, *MMP* gene expression is presented as heat map for each cell type in LV tissue, indicating that *MMP* expression is highly variable in different cell types. In non-diseased LV tissue, *MMP-2* is predominantly expressed by fibroblasts, in line with in vitro studies [11, 71], while other MMPs like *MMP-14*, *-16* or -*24* are almost equally expressed by several cell types.

Besides providing gene expression data, the GTEx project also built a catalogue of genetic effects on gene expression across different tissues and identified genomic variants that influence the gene expression, so-called expression quantitative trait loci (eQTLs) [39]. Genomic variants such as single-nucleotide polymorphisms are capable of altering all steps of gene expression in dependency of their genomic position, but polymorphisms in transcriptional regulatory elements in particular are known to influence the mRNA levels. Both, genotype data from whole-genome sequencing together with RNA-sequencing data were available from 386 donors for LV tissue identifying 9642 genes significantly regulated by genetic variations (eQLT genes) and from 213 donors for coronary artery tissue identifying 6296 eQTL genes [39]. Out of these identified genes, we listed MMP coding genes with at least one genetic variation that influences their transcription in LV or coronary artery tissue (Table 1). For example, while 93 eQTLs are described for *MMP-1* in LV-tissue, none of the variants affect *MMP-1* expression in coronary arteries.

**Table 1:** List of MMP genes with at least one significant cis-eQTL in left ventricular or coronary artery tissue according to the GTEx data source and their respective numbers of eQTLs

		Left ventricle	Coronary artery
Total samples with donor genotype		386	213
Number of significant eQTL genes^a^		9642	6296
Gene symbol	Gencode Id	Numbers of eQTLs	
*MMP-1*	ENSG00000196611.4	93	–
*MMP-7*	ENSG00000137673.8	–	3
*MMP-11*	ENSG00000099953.9	206	–
*MMP-16*	ENSG00000156103.15	398	162
*MMP-17*	ENSG00000198598.6	29	–
*MMP-21*	ENSG00000154485.4	–	4
*MMP-23A*	ENSG00000215914.4	20	3
*MMP-25*	ENSG00000008516.16	27	–
*MMP-28*	ENSG00000271447.5	5	–

^a^eQTL genes are genes with at least one significant *cis*-eQTL acting upon them. Data source: GTEx Analysis Release V8 (dbGaP Accession phs000424.v8.p2) on 19.04.2021 [39]

In Fig. 2c, GTEx data for *MMP-16* are shown exemplarily: RNA-sequencing coverage as well as eQTL data for coronary artery tissue are depicted in the upper panel and for LV tissue in the lower panel. The *MMP-16*-eQTLs are indicated as red dots with their *p*-value on the *Y*-axis—162 eQTLs in coronary and 398 eQTLs in LV tissue. The RNA-sequencing coverage shows a higher expression of *MMP-16* in coronary artery tissue (light red) than in LV tissue (purple). Furthermore, RNA-sequencing counts correspond well to the exon structure of the *MMP-16* gene depicted below. Although GTEx shows an association between polymorphisms and gene expression level, this by itself does not establish clinical relevance. Therefore, a reasonable complement are clinical cohort or genome-wide association studies (GWAS), which may reveal a relationship between disease development, progression, or outcome, and specific polymorphisms, but usually cannot examine gene expression in cardiac or coronary tissue.

## MMPs in human cardiovascular diseases

### Atherosclerosis and CAD

#### MMP expression in atherosclerosis and CAD

**Fig. 3:**
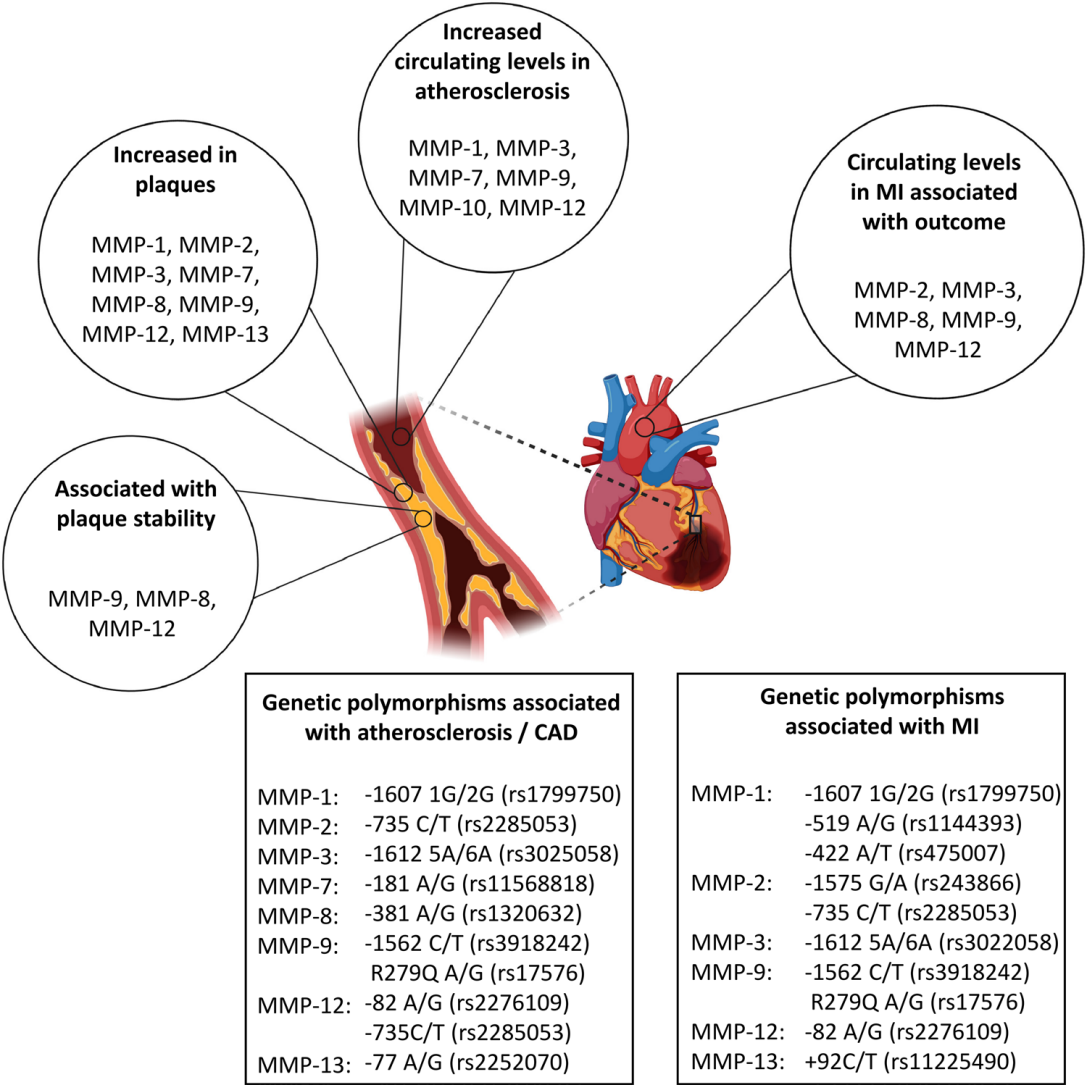
Matrix metalloproteinases in atherosclerosis and myocardial infarction. Various matrix metalloproteinases (MMPs) are increased in atheriosclerotic plaques and are involved in plaque stability. Additionally, circulating levels of some MMPs are increased in atherosclerosis and myocardial infarction (MI). The boxes show MMPs and respective single-nucleotide polymorphisms, which are shown to be associated with atherosclerosis / coronary artery disease (CAD) or MI in at least one study

For more than 20 years, MMPs have been studied in human atherosclerotic plaques. In most studies, increased abundance of the investigated MMPs, such as MMP-1, -2, -3, -7, -8, -9, -12, and -13 was found in vulnerable regions of human atherosclerotic plaques as summarized in Fig. 3 [33, 40, 43, 86, 113]. Moreover, some of these studies examined not only the presence but also the activity of MMPs revealing that the activity of MMP-1, -2, -3, and -9, measured by in situ zymography, was upregulated in the plaques [33]. Interestingly, regions with higher MMP activity were prone to plaque rupture [33]. Partially conflicting results were reported by Molloy et al. In their study, the active levels of MMP-1, -13 and -8 were quantified by ELISA, but only the active form of MMP-8 was upregulated in plaques, whereas the levels of active MMP-1 and MMP-13 were not altered [86]. Higher activity of MMP-8 as well as of MMP-9 was confirmed in rupture-prone plaques whereas MMP-2 activity was increased in fibrous, more stable plaques [111]. Studying MMP expression on cellular level revealed that the expression of *MMP-1, -3, -7, -8, -12* and *-13* was mostly attributed to macrophages [1, 33, 40, 80, 86, 113]. In addition, MMP-1, -3, and -9 were also detected in smooth muscle cells (SMCs) and lymphocytes [33, 127], while MMP-1 and MMP-10 were co-localized with plaque endothelium [33, 87]. In a published single-cell sequencing dataset of human arteriosclerotic plaques, *MMP-2* and *-28* were detected as marker genes for a cluster of endothelial cells representing a type of activated endothelium, which might exacerbate inflammation through cell adhesion, neovascularization and leukocyte extravasation. *MMP-9* and *MMP-19,* on the other hand*,* were found to be marker genes for a cluster of myeloid CD68-positive cells that exhibited a foam cell phenotype [24]. Thus, the increased MMP-8 and MMP-9 activities detected in plaques could be a reflection and subsequent risk factor of dysregulated inflammation.

The association between MMP expression or activity with plaque instability has been investigated in several studies, the results are summarized in Fig. 3. Among them, the correlation of increased MMP-9 expression or activity with an unstable plaque phenotype has been repeatedly described [43, 75, 99], and increased MMP-8 expression has also been associated with an unstable plaque phenotype [99, 111]. Additionally, MMP-12 has been associated with symptomatic atherosclerosis [80]. The authors suggested that MMP-12 activity influences plaque progression through elastin degradation and macrophage invasion. In contrast, more contradictory results have been reported for MMP-2: while Heo et al. associated high *MMP-2* expression with cap rupture, intra-plaque haemorrhage, and a thin fibrous cap [43], Sluijter et al. found increased MMP-2 activity in stable lesions associated with the presence of SMCs and a fibrous phenotype [111]. In a third study examining MMP-2 activity in plaques, as with MMP-1 and MMP-3, no differences were found between symptomatic and asymptomatic patients [75]. Since molecular study of human plaques is limited by their low availability, many studies investigate the influence of MMPs on plaque stability in atherosclerosis mouse models. Here, the accumulation of SMCs in the cap of the arteriosclerotic lesions was frequently investigated, as it has an impact on plaque stability: it was shown, that the presence of MMP-2, -3 and -9 resulted in more SMCs in plaque lesions, indicating more stable plaques and a beneficial role of these MMPs [59, 67]. The opposite effect, thus the knock-out leading to an increase in SMCs and consequently higher plaque stability, was shown for MMP-7 and MMP-12 [59]. Additionally, MMP-7 promoted vascular SMC apoptosis, which could affect plaque stability [136]. This detrimental role in plaque stability, was also shown for MMP-14. Its upregulation in macrophages of ApoE-deficient mice was accompanied by slight decrease in vascular SMC content of the lesions [25]. Another indicator of plaque stability is a high collagen content. In this regard, MMP-deficiency appears to be beneficial. In ApoE-deficient mice, knock-out of MMP-8 as well as MMP-13 resulted in increased amounts of collagen in lesions or the intima [68]. Also in another study MMP-13-deficiency lead to an increased collagen amount in plaques, indicating a more stable phenotype, while it was not shown to participate in plaque formation [22]. Although differences in collagen content were not found in MMP-12-deficiency, its absence protected against elastin degradation, also indicating more stable plaques [78].

In addition to examining MMP activity and expression in atherosclerotic plaques, several studies determined serum or plasma levels of MMPs and correlated them with plaque burden, plaque development, or outcome (Fig. 3). Thus, the levels of MMP-1, -3, -7, -10 and -12 were increased in patients with carotid atherosclerosis [1, 47, 94]. Moreover, the levels of MMP-1, -3, and -12 were significantly positively correlated with cardiovascular and cerebrovascular events in patients with carotid atherosclerosis [47]. In case of MMP-12, this was confirmed in another study [80] and for MMP-1 and MMP-3 additional studies have associated their circulating levels with the presence of atherosclerotic lesions [7] or MMP-1 levels with total plaque burden [69].

To assess a relationship between MMPs and plaque development, a number of studies investigated intima-media thickness (IMT) and circulating MMP levels. The plasma levels of MMP-1, -3 and -7 were elevated in a random cohort of participants with high IMT values compared with a random cohort with low IMT, while MMP-2, -8 and -9 were not increased in the same study [35]. This result was confirmed for MMP-9 in other studies in which circulating levels were not associated with increased IMT [7, 94]. However, contradictory results have been reported for MMP-1 and MMP-3 levels, with no IMT association in patients with dyslipidemia or cardiovascular risk factors [7, 94]. Levels of circulating MMP-9 were significantly higher in patients with a fatal cardiovascular event during follow-up than in survivors [10]. In contrast, MMP-12 plasma levels were associated with IMT progression in patients at high risk of CVD [80]. Thus, multiple studies indicate altered circulating levels of MMPs in arteriosclerosis and CAD. However, the results for specific MMPs appear contradictory which indicate study, disease and cohort specific contextuality. It also highlights challenges to draw conclusions when the pathophysiological mechanisms underlying the changes are incompletely elucidated. This impression is further reinforced by the different intra- and extracellular functions of MMPs.

#### Genetic polymorphisms of clinical relevance

As described above, polymorphisms within the promotor region or even in the coding sequence of *MMP* genes can affect their gene expression. Thus, associations between polymorphisms and CAD were investigated in a number of studies.

The 2G-allele of the MMP-1 polymorphism rs1799750 (-1607 1G/2G) was associated with the presence of femoral plaques, but not carotid plaques, in participants with mainly non-severe stenotic plaques [97]. For MMP-3 it has been shown that the polymorphism rs3025058 (-1612 5A/6A) affects the MMP-3 promoter activity thereby regulating the *MMP-3* expression [141]. While, the 6A-allele was associated with reduced *MMP-3* expression and linked to the progression of atherosclerosis and CAD [51, 110, 140–142], it is suggested that the 5A allele is beneficial in atherosclerosis. Consequently, as MMP-3 is an anti-adipogenic factor, these studies propose that therapy of CAD in 6A allele carriers should focus on intense lipid-lowering programs. Nevertheless, a potential link between the polymorphisms, MMP-3 and CAD is more intricate—a meta-analysis of multiple studies of rs3025058 found that European participants carrying the 5A allele had a reduced, but East Asian participants with the 5A allele had an increased risk of developing MI [64]. In another study, specific combinations of the MMP-1 polymorphism rs1799750 and the MMP-3 polymorphism rs3025058 (2G/1G&6A/6A, 2G/1G&6A/5A, 2G/1G&5A/5A, 1G/1G&5A/5A) were associated with CAD compared to 2G/2G&6A/6A genotype in a univariate analysis [46]. Contrarily, 2G/2G genotype in combination with 6A/6A genotype was found to predict an increased risk of internal carotid artery stenosis and the 6A/6A genotype alone was an independent risk factor of carotid stenosis [38].

The polymorphism rs11568818 in the *MMP-7* promotor region (-181 A/G) has been reported to play an important role in the development of vulnerable plaques. The frequency of A/G and G/G genotypes was significantly higher in patients with vulnerable plaques, and rs11568818 was also associated with vulnerable plaques independently of other factors [48]. In other studies, the same genotypes were more prevalent in femoral but not in carotid plaques [97], and G-allele carriers were shown to have a smaller coronary artery luminal diameter [60].

Two polymorphisms in the *MMP-8* promotor, rs11225395 (-799 C/T) and rs1320632 (-381 A/G) were analysed in the context of carotid plaques. Thereby, the -381G allele resulted in higher mRNA expression of *MMP-8* in the plaque. However, this was associated with a higher incidence of carotid atherosclerosis only in female patients, while MI susceptibility or plaque stability was not assessed in this study [26].

The MMP-9 polymorphism rs17576 (R279Q A/G in exon 6) was associated with the presence of plaques in femoral and carotid arteries in male patients [97], while in another study rs17576 was associated with MI but not with CAD [46]. In a large meta-analysis involving more than 10,000 CAD patients ethnic differences in association with CAD were found for the MMP-9 polymorphism rs3918242 (-1562 C/T): while East Asian T-allele carriers had an increased risk of CAD, no significant difference was found in Western populations [132]. Still, in some of the included studies with Western participants, T-allele carriers were found to have an increased risk of cardiac events during CAD [93] or an increased risk of developing coronary artery stenosis [88]. In addition, C/T and T/T genotypes were significantly associated with the mean area of complicated plaques, and the genotype was an independent predictor of complicated lesion area after adjustment in patients ≥ 53 years of age [101].

Associations between polymorphisms of other MMPs and CAD have rarely been investigated. In a Chinese cohort, an association was found between the MMP-2 polymorphism rs2285053 (-735 C/T) and the formation of vulnerable plaques in the carotid artery, with the T/T genotype appearing to be protective and overrepresented in cases with stable plaques [128]. The MMP-12 polymorphism rs2276109 (-82 A/G) was associated with the occurrence of femoral plaques in women but not in men [97]. The MMP-13 polymorphism rs2252070 (-77 A/G) showed a significant correlation to fibrous plaques in the abdominal aorta in young black male participants [143].

### Myocardial infarction and heart failure

#### MMP expression in the context of MI

Cardiac tissue samples of patients suffering from MI are rarely available. Therefore, most studies investigating MMP expression in the context of MI focus on circulating and plasma levels of MMPs to investigate MMPs as predictors for LV remodelling and outcome, which are summarized in Fig. 3.

In two studies, plasma levels of MMP-2 were elevated in patients with acute MI compared to controls with CAD [62, 70]. In another study, MMP-2 levels were not found to be elevated in patients with acute MI compared with control subjects with stable CHD, yet the control group consisting of 15 participants was very small compared with the other studies [95]. Webb et al. even reported downregulated MMP-2 levels after MI compared to age-matched healthy controls [133]. Interestingly, Squire et al. demonstrated higher MMP-2 plasma levels in inferior compared to anterior MI, which was not considered in the other studies [112]. Several studies reported on elevated MMP-9 levels after MI [55, 62, 95, 133]. Additionally, Webb et al. reported on elevated MMP-8 levels after MI, while MMP-7 was not altered compared to age-matched healthy controls [133]. MMP-12 plasma levels were elevated in patients with MI with ST elevation compared to patients with stable angina pectoris as well as healthy controls [129].

Several studies not only examined changes in plasma levels but also associated them with outcome after MI. Nilsson et al. reported that circulating MMP-2 levels 0–12 h after MI were negatively correlated with LV function and positively correlated with infarct size at 4 months of follow-up [91]. Contrarily, Squire et al. reported on a significant inverse correlation between circulating MMP-2 levels and LV volume, while MMP-9 positively correlated with LV volume [112]. Another study demonstrated an association between circulating MMP-2 and MMP-9 activity after MI and increased LV end-diastolic and systolic volumes after a follow-up period of 6 months [84]. Also in mouse models of MI, several studies showed that the deficiency of MMP-2 as well as MMP-9 protected mice from cardiac rupture, accompanied by attenuated LV dilation and less macrophage infiltration [23, 27, 42, 45, 83]. Contrarily, a deficiency of MMP-12 or MMP-28 aggravated cardiac function and reduced survival due to an increase in cardiac rupture post-MI in mice. The improved survival of wildtype compared to MMP-12-deficient mice was attributed to higher expression levels of MMP-12 produced by Ly6C^low^ macrophages possibly through promoting wound healing and reducing neutrophil infiltration [65, 79]. Ventricular wall rupture in MMP-28-deficient mice resulted from a defective inflammatory response and insufficient scar formation indicated by reduced mRNA expression of pro-inflammatory and pro-fibrotic genes [79]. In human, elevated MMP-3 plasma levels were determined in the course of acute MI and were associated with reduced ejection fraction, indicating worsened LV dysfunction, recurrent acute MI and an increased risk of death [63]. In line with this, Cavusoglu et al. found that plasma MMP-3 levels were an independent predictor for MI until 5 years of follow-up [14]. An association between plasma level and clinical outcome was also reported for other MMPs. Persistently elevated MMP-9 levels 5 days after MI were accompanied by a threefold end-diastolic volume increase at day 28 [133]. In another study, maximal MMP-9 levels were predictive for lower LV ejection fraction at admission and for greater changes in LV end-diastolic volume between admission and follow-up, while high MMP-9 levels during follow-up period were associated with relative preservation of LV function [62]. In contrast to these studies, Jefferis et al. only found a univariate association of MMP-9 serum levels with MI and stroke, while those were not an independent risk marker [55]. Accordingly, baseline levels of MMP-2, -8 and -9 were positively associated with cardiovascular death or hospitalization for heart failure by a univariate analysis, while only MMP-8 baseline levels were an independent predictor of LV remodelling and cardiovascular outcome after MI [29].

**Fig. 4:**
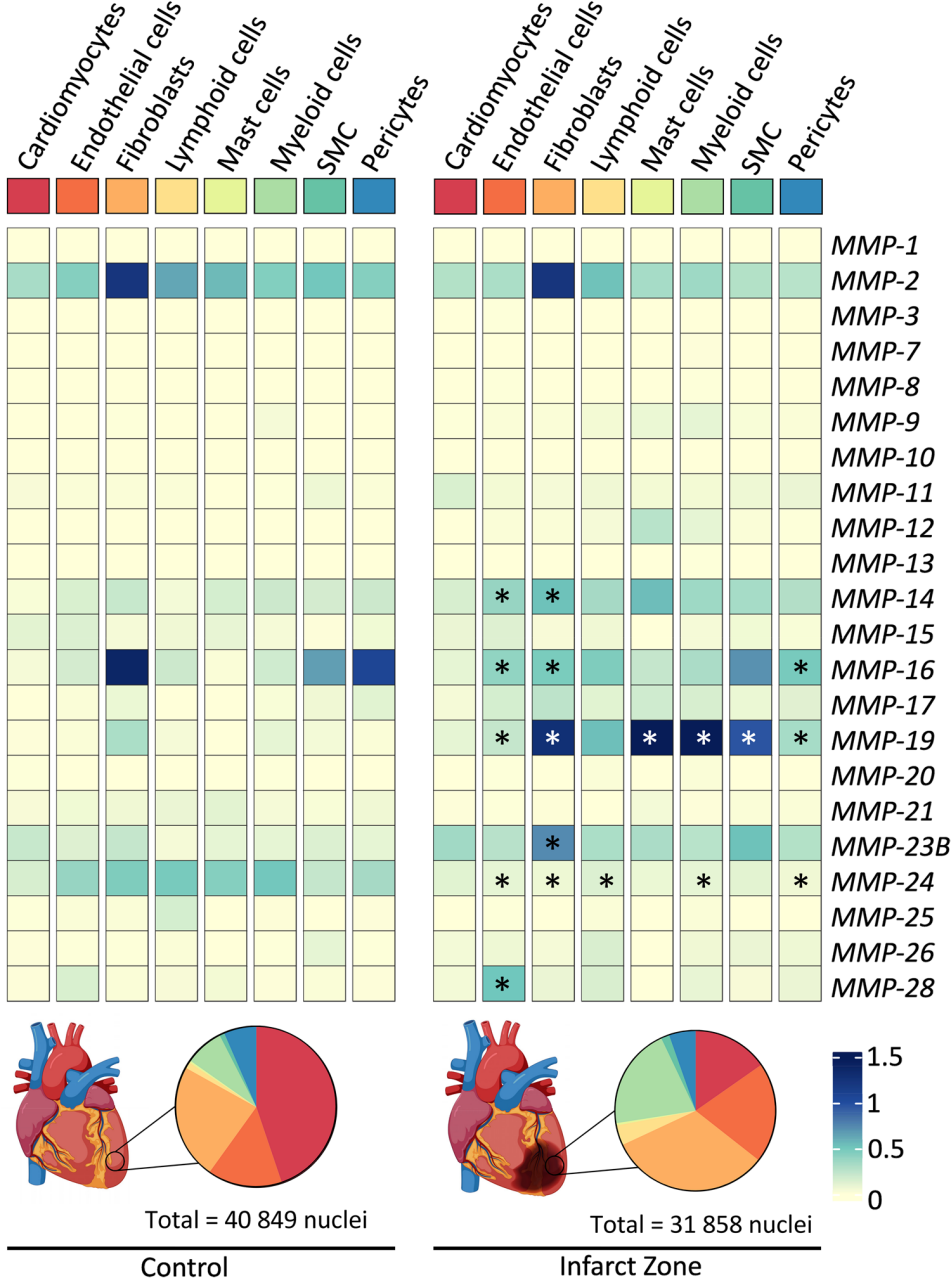
*MMP* expression on single-cell level in the infarct zone after myocardial infarction in human hearts [66]. Average gene expression was calculated and compared between controls and ischaemic hearts for each cell type and plotted as heat map. Differential gene expression between control and infarct zone was calculated with the FindMarker function of Seurat (* = adjusted *p*-value < 0.05). Cell populations in the control as well as in the infarct tissue were plotted as pie charts. Note that adipocytes, neuronal and cycling cells were excluded from this analysis

A more newly developed method—single-nuclei sequencing—allows to study gene expression at the cellular level even from frozen tissue and therefore improves the opportunities to study the expression of *MMPs* in the context of MI. Recently, one such dataset was published that examined cellular gene expression from more than 20 hearts, including 4 non-transplanted donor hearts and samples of necrotic areas from 12 patients with acute MI [66]. Samples from this infarct zone were collected from all patients at various time points (2–45 days) after the onset of clinical symptoms, before the patients received an artificial heart or an LV assist device. Using the publicly available dataset, *MMP*-expression can be compared in each cell type between the control tissue and the infarct zone (Fig. 4). Note that there are significantly fewer nuclei in the infarct zone than in the control tissue and that the proportions of cell types diverge greatly between these groups. For example, in the control tissue 45% of the cells are cardiomyocytes, whereas in the infarct tissue only 15% of the cells are cardiomyocytes as shown in Fig. 4 [66]. This might be an explanation, why cardiomyocytes showed no differences in *MMP* expression in the infarct zone after MI, while expression of *MMP-14*, *-16*, *-19*, *-23B* and *-28* was increased in other cell types. Contrarily, *MMP-24* expression was downregulated in most cell types. Overall, only few *MMPs* were differentially regulated after MI or were below the detection limits, which are relatively high in single-nuclei sequencing approaches [66]. However, it should be noted that this analysis is based on mRNA levels. Since MMPs are enzymes, a very small amount of activated protein in stressed cells can have considerable effects, so mRNA levels alone are not sufficient to determine the importance of MMPs in MI.

#### Genetic polymorphisms of clinical relevance

Several studies investigated the relationship between different MMP polymorphisms and MI or heart failure, summarized in Fig. 3. The MMP-1 polymorphism rs1799750 (1G/2G) is often related to MI and cardiovascular death. In a small study with 42 patients, participants with 2G/2G genotype had an increased risk for LV remodelling after MI compared with other genotypes [82] and were associated with an increase in QRS widening rate [92]. In contrast, it is described that the 2G polymorphism is related to higher transcription rates of MMP-1 in fibroblasts [108], which was shown to lead to substantially reduced heart failure-related mortality [125]. However, in heart failure patients, an ischemic etiology with previous MI and regional LV dysfunction was more frequent in MMP-1 2G allele carriers in the same study [125]. Other studies did not find differences in distribution of 1G/2G genotypes in patients with MI history or acute MI and control group [37, 105]. Additionally, the polymorphism was not associated with the combined endpoint in a Caucasian cohort study [98]. However, in this study other MMP-1 polymorphisms rs1144393 (-519 A/G) and rs475007 (-422 A/T) showed significant association with combined endpoint after MI [98].

The AA genotype of the polymorphism rs243866 (-1575 G/A) in the promotor region of MMP-2 was associated with developing MI in a Mexican population and more frequent in patients with MI [100]. However, outcome after MI was not associated with rs243866 in another study [98]. For another polymorphism in the MMP-2-promotor, rs2285053 (-735 C/T), the frequency of the TT genotype was significantly lower in controls than in MI-patients compared with the CC genotype in a Turkish population [4]. Still, this could not be confirmed in another study [100]. Also for other MMP-2 polymorphisms rs243865 (-1306 C/T) and rs243864 (-790 G/T) associations with MI or heart failure were not detected [4, 46, 85, 92, 100].

The polymorphism rs3025058 in the promotor region of MMP-3 (-612 5A/6A) was associated with MI in several studies. Thereby, the 5A/5A genotype was significantly more frequent in patients with acute MI than in controls and logistic regression analysis indicated that the 5A allele is an independent risk factor for the development of MI [37]. Another study showed similarly that the prevalence of the 5A allele was significantly more frequent in patients with MI than in controls and additionally an independent risk factor for acute MI [117]. Moreover, the 5A/5A genotype was associated with cardiac mortality in patients with non-ischemic heart failure [85]. In contrast, no differences in the distribution of the polymorphism between the MI group and controls were observed in a Mexican population [105] and it was described as being protective against QRS widening [92] suggesting reduced cardiac remodelling.

Only one study reported on significant differences in allele frequency in rs11568819 (-153 C/T) of MMP-7 in MI patients versus controls [5], whereas others reported on similar frequencies in rs11568819 [100] as well as rs115688198 (-181 A/G) [98, 100].

Combined MMP-9 polymorphisms rs3918242 (-1562 C/T) and rs17576 (R279Q A/G in exon 6) were associated with an increased risk of MI in a Caucasian study cohort [46]. Thereby, the CT/RQ and TT/QQ genotype were significantly associated with MI incidence [46]. Both polymorphisms were investigated independently in other studies [85, 92, 98, 105, 125, 130]: here, rs17576 was not associated with MI [130]. For the polymorphism rs3918242, the T/T genotype was more frequent in the MI group than in controls and additionally the C/T and T/T genotype were identified as independent risk factors for MI [130]. This was confirmed in a Mexican cohort, where C/T and T/T genotypes were associated with increased risk of developing MI compared to C/C genotype [105]. Furthermore, the T allele was an independent predictor of cardiac mortality in heart failure patients [85] and the T/T genotype was reported to have a trend to affect disease progression and long-term survival after MI [98]. In contrast, rs3918242 was not associated with QRS widening rate in patients with heart failure [92] and the outcome in Brazilian heart failure patients [125].

In one study analysing the MMP-12 polymorphism rs2276109 (-82 A/G), carriers of the AG or GG variants showed an increased risk of a higher number of occlusions in their coronary arteries [100]. Still, no associations were found with LV dysfunction and heart failure [92, 98]. For the MMP-13 polymorphism rs11225490 (+92 C/T) an association with combined endpoint and a worse outcome after MI for CC carriers was shown [98].

## Inhibitors of MMPs as potential new therapeutics

As described in the previous sections, higher levels or activity of MMPs are often associated with cardiac diseases and are thus may represent a potential treatment option. The early MMP inhibitors were all broad-spectrum inhibitors, usually targeting the catalytic Zn^2+^ ion [30, 135]. These have been tested in clinical trials for the treatment of various cancers, but have all failed due to side effects that are likely attributable to the inhibitors’ lack of specificity [124]. Improved broad-spectrum inhibitors of MMPs have also been tested in cardiac disease in preclinical trials in in-vivo animal models and in a few cases of patients, which will reviewed in the following section.

In an atherosclerosis model in mice, the MMP inhibitor RS-130830 aggravated plaque formation and stability [58]. Treatment of LDL receptor- or apolipoprotein E (ApoE)-deficient mice with the non-selective MMP inhibitor CGS 27023A had no beneficial response on plaque development [102]. Similar results were observed by treating ApoE-deficient mice with doxycycline, another broad-spectrum inhibitor preferentially inhibiting MMP-2 and MMP-9 [81]. The MIDAS prospective, double-blind placebo pilot study evaluated the effect of subantimicrobial doxycycline in angina patients. Since this study was likely underpowered, it failed to detect a difference in the composite endpoint, including fatal or nonfatal MI. However, it did show that treated patients had lower levels of C-reactive protein and interleukin-6, suggesting a beneficial effect on the inflammatory response [12].

The effects of doxycycline on remodelling after MI have been investigated in several animal studies. In rats, early MMP inhibition by doxycycline after MI lead to a preservation of LV structure and passive function and also late and long-term administration (2–7 days post-MI) improved LV structure after MI [34, 126]. In line with this, Camp et al. showed that continuous administration of doxycycline from 2 days before MI to 4 weeks after MI decreased infarct size, improved cardiac fibrosis and cardiac conductance in rats [13]. Only one study revealed contradictory results: even if doxycycline treatment decreased MMP-2 and -9 activity after MI it did not prevent LV remodelling or dysfunction [118]. The phase II trial TIPTOP analysed doxycycline in addition to standard therapy in patients with acute STEMI and LV dysfunction. Doxycycline treatment significantly reduced LV remodelling and the rate of death, MI, congestive heart failure and stroke in a 6 months follow-up [15]. Another broad-spectrum MMP inhibitor GM6001/ilomastat reduced infarct size when administered either before the onset of ischemia or the onset of reperfusion in rats and mice [8, 9]. Despite the positive results of preclinical studies and small clinical trials for the treatment of atherosclerosis or to improve remodelling after MI, non-selective MMP inhibitors are still not approved for the clinic due to the lack of large phase III trials.

To overcome challenges with broad-spectrum MMP inhibitors, more selective MMP inhibitors were developed. The inhibitor CP-471,474 selectively targets MMP-2, -3, -9 and -13 rather than MMP-1. After experimental MI, CP-471,474 did improve cardiac function in mice and rabbits [73, 106]. In pigs, PGE-530742 (renamed to PG-116800, inhibiting MMP-2, -3, -8, -9, -13, and -14, sparing MMP-1 and -7) as well as PD166793 (inhibiting MMP-2, -3 and -13 sparing MMP-1, -7 and -9) attenuated LV remodelling after MI [89, 139]. In case of PD166793, even infarct size was decreased 2 weeks after MI [89]. Oral administration of PG-116800 was even tested in the phase II double-blinded PREMIER trial in patients suffering from MI. Yet, the inhibitor had no beneficial effects on LV remodelling or outcome after MI in patients. This could be explained by the low concentrations used, which were below the reported effective dose. But still, adverse events like arthralgia and joint stiffness were increased [49].

Besides the selective MMP inhibitors targeting not all but multiple MMPs, several pharmaceutical substances exist that target only one MMP. However, specific inhibition of MMPs in cardiac conditions was not yet as successful as with broad-spectrum and selective inhibitors. Inhibition of MMP-9 early after inducing experimental MI in mice aggravated cardiac function, because immune responses were prolonged, contradicting results gained from studies with MMP-9 knock-out mice [52]. The highly selective MMP-12 inhibitor RXP470.1 exacerbated LV dysfunction, in line with the fact that MMP-12 was already described to be protective after MI [53]. In atherosclerosis, however, that same MMP-12 inhibitor was beneficial, confirming disease-specific effects of MMPs [57]. Pharmacological inhibition of MMP-2 with the selective inhibitor TISAM increased survival after experimental MI in mice compared to non-treated mice. The survival rate was similar to that of MMP-2 knock-out mice with only approximately 20% mortality compared to 50% mortality in untreated mice [83]. However, data included in the publication show that other MMPs are also inhibited by TISAM, albeit with slightly lower binding affinity, so one might question whether TISAM is truly a specific inhibitor.

## Conclusion and future perspectives

Analyses of published and publically available data support MMPs as promising targets in the treatment of atherosclerosis and MI. Nevertheless, multiple challenges exist in regard to the incomplete knowledge of cellular sources as well as tissue-specific and systemic consequences of distinct MMP activities in the context of atherosclerosis and MI. For research in this field, new techniques such as single-cell or single-nucleus sequencing offer new opportunities to study the expression profiles of MMPs at a cellular level in healthy or diseased human tissues. Since the activity of MMPs is strongly regulated at the protein level, analysis of MMPs only at the mRNA level may never be sufficient, but these new data may complement previous research. As the number of available online datasets continues to increase, this review also shows how these data can be processed and used to address the specific question of MMP regulation in cardiac disease. In addition, the GTEx database provides the opportunity to gain insights into the association between polymorphisms and gene expression. Furthermore, clinical studies demonstrated the correlation between polymorphism and disease burden in many cases. Further insights into the relationship between polymorphisms, altered MMP expression, and clinical outcome may be an important approach to more individualized cardiovascular disease therapy in the near future. Initial attempts to pharmacologically inhibit MMPs have shown promising results in animal models of atherosclerosis and MI as well as in first human clinical trials, although larger clinical studies are needed in the future.

## Data Availability

Not applicable.
